# The Application of Hydrogen Sulfide Fluorescent Probe in Food Preservation, Detection and Evaluation

**DOI:** 10.3390/molecules29163973

**Published:** 2024-08-22

**Authors:** Sitong Chen, Xiongjie Zhao, Liyi Zhou

**Affiliations:** 1 College of Food Science and Engineering, Central South University of Forestry and Technology, Changsha 410004, China; 2 College of Chemistry and Biological Engineering, Hunan University of Science and Engineering, Yongzhou 425199, China

**Keywords:** hydrogen sulfide, foods, fluorescence probe, food preservation

## Abstract

This work primarily reviewed the response mechanism of fluorescent probes for H_2_S detection in foodstuffs in recent years, as well as the methodologies employed for detecting foodstuffs. Firstly, the significance of studying H_2_S gas as an important signaling molecule is introduced. Subsequently, a review of the response mechanism of the scientific community on how to detect H_2_S in foodstuffs samples by fluorescent probe technology is carried out. Secondly, the methods commonly used for detecting foodstuffs samples are discussed, including the test strip method and the spiking recovery methods. Nevertheless, despite the significant advancements in this field, there remain some research gaps. Finally, the article identifies the remaining issues that require further attention in current research and proposes avenues for future investigation. More importantly, this work identifies the current limitations of research in this field and proposes future applications of fluorescent probes for H_2_S in assessing food freshness and determining food spoilage. Therefore, this review will provide robust technical support for the protection of consumer health and the advancement of the sustainable development of the food industry and also put forward some new ideas and suggestions for future research.

## 1. Introduction

For decades, hydrogen sulfide (H_2_S) has been considered a toxic gas with a rotten egg smell. However, recent studies have revealed the key role of H_2_S as an important gas signaling molecule and redox balance molecule in living organisms. Produced naturally from L-cysteine, cystathionine γ-lyase (CSE) and cystathionine β-synthase (CBS) [1], H_2_S acts as a gaseous signaling molecule and plays important roles in several physiological systems. In the nervous system, H_2_S is involved in neurotransmission processes, regulating NMDA receptors and TRP channels through S-mercapturization modifications [2], and influencing intracellular calcium ion concentrations and neurotransmitter transmission [3]. In the cardiovascular system, H_2_S has a significant antihypertensive effect, mainly through inhibition of the renin–angiotensin system, inhibition of vascular remodeling, and direct diastole of the vascular smooth muscle [4]. In addition, H_2_S is involved in cell proliferation [5], apoptosis regulation [6], and antioxidant [7] and anti-inflammatory [8] physiological processes. However, abnormal levels of H_2_S in the body can also cause several health problems, such as Down syndrome, Alzheimer’s disease, cirrhosis of the liver, and atherosclerosis [9].

In addition, H_2_S is inextricably linked to most high-protein foods [10]. As one of the major volatiles produced by spoiled foods, H_2_S concentration is commonly used as an indicator of the freshness of high-protein foods. For example, yeasts in wine produce H_2_S gas, which can negatively affect the sensory quality of fermented foods and beverages [4]. Excessive concentrations of H_2_S can cause quality degradation of alcoholic beverages [11,12], resulting in economic losses or serious food safety problems. Therefore, the detection of H_2_S levels in real food samples is of great importance, and the development of a faster and more convenient method for detecting H_2_S in real food samples has become particularly important.

Fluorescence imaging has become a fundamental tool for detecting gas molecules, renowned for its non-invasive, real-time imaging capabilities, high sensitivity, and high spatial and temporal resolution. Concurrently, its exemplary biocompatibility, high selectivity, non-destructive analytical attributes, and straightforward operation have established it as a principal analytical methodology for biomolecular cellular observation and food monitoring [13,14,15,16,17]. As a result of ongoing research, a variety of fluorescent probes for the detection of H_2_S have been developed and synthesized. These include probes based on the azide/nitro reduction reaction [18], nucleophilic reaction [19,20], substitution reaction [21], strong S-Cu bonding ability [22], binding to unsaturated double bonds [23], and metal sulfide precipitation reaction [24].

It is noteworthy that MOF, as a novel material, can also be employed in the fabrication of biochemical sensors with H_2_S detection probes. Metal–organic frameworks (MOFs) are a novel class of porous crystalline hybrid materials comprising metal ions and organic linkers. They exhibit high porosity and biodegradability, which are advantageous for various applications [25]. The combination of MOFs and a fluorescent probe has the potential to significantly enhance its sensitivity. Additionally, MOFs are frequently employed in the development of biosensors. The fundamental principle underlying this biosensor design methodology can be elucidated as follows: The initial step involves reducing the substance under examination to generate an electrochemical signal. Secondly, the substance under examination reacts with the substance to be tested, resulting in a change to its molecular structure. This, in turn, gives rise to a luminescent phenomenon. Thirdly, certain substances themselves emit fluorescence. When they react with the substance under examination, they produce a fluorescence enhancement phenomenon. The specific substances employed for detection are contingent upon the analyte in question [26]. MOF structures are particularly well-suited for detecting hydrogen sulfide in food products. However, the number of fluorescent probes for detecting H_2_S in food remains relatively limited.

**Scheme 1:**
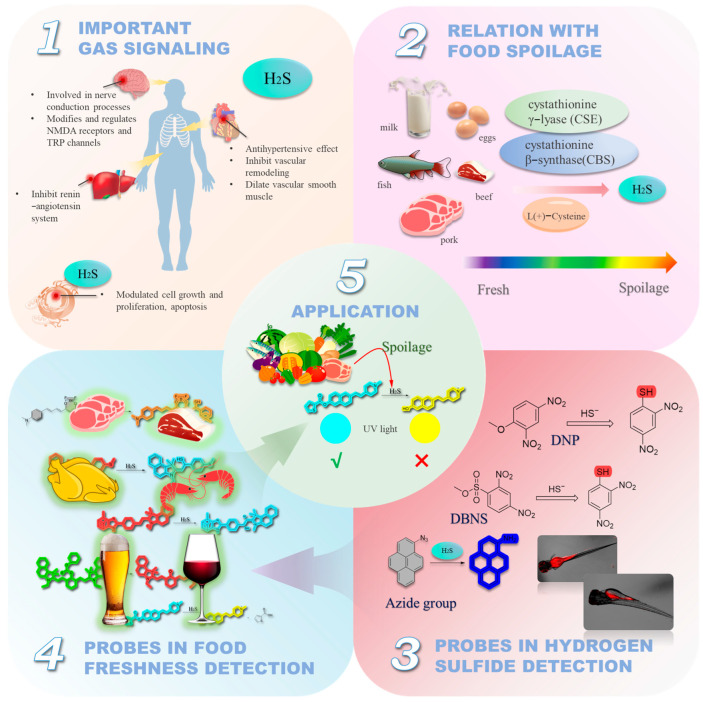
Formation and action of H_2_S and its application to fluorescent probes.

This review examines the fluorescent probes designed for detecting H_2_S in real food samples (Scheme 1). These probes employ a variety of chemical reactions, including the 2,4-dinitrophenyl (DNP) thiolysis reaction, the double bond addition-based 7-nitrobenzo-2-oxa-1,3-diazole (NBD) thiolysis reaction, and the azide-based reaction. Furthermore, two prevalent techniques employed in the analysis of food samples are elucidated, and the document additionally contemplates the prospective utilization of H_2_S fluorescent probes in the evaluation of food safety, particularly in the identification of food preservation and deterioration characteristics. In conclusion, this review offers researchers a comprehensive overview of the latest developments in the design and application of fluorescent probes for food testing. Additionally, it provides food safety experts with a valuable repository of fundamental research and innovative concepts. The implementation of enhanced detection techniques for H_2_S in food samples, coupled with the expedient identification of any contamination, can markedly enhance the overall safety of the food supply chain.

## 2. Fluorescent Probes for the Detection of H_2_S in Foods

The synthesis of a variety of fluorescent probes has enabled the development of methods for detecting hydrogen sulfide (H_2_S) in real food samples. Moreover, the recognition group of H_2_S determines whether the fluorescent probe can be responded to and influences the response time. The recent fluorescent probes for detecting H_2_S in food can be classified into the following five categories.

### 2.1. Thiolysis Reactions Based on 2,4-dinitrophenyl (DNP)

**Figure 1:**
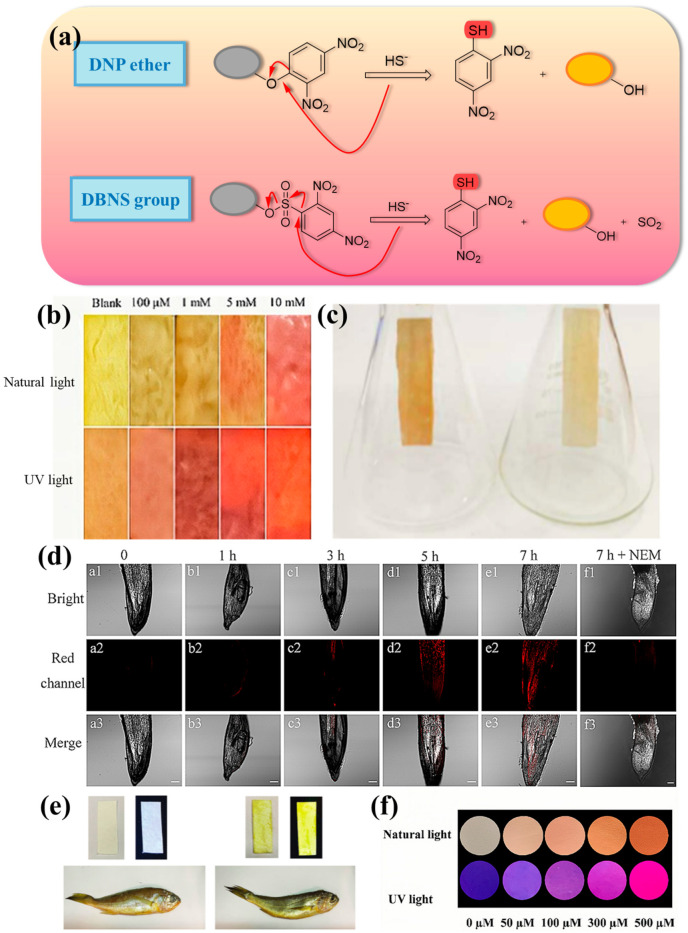
(**a**) Approximate response mechanism of DNP ether and DBNS group [35]. (**b**) Colorimetric (top) and fluorochrome changes (bottom) of probe **7** test strips immersed in different concentrations of H_2_S [27]. (**c**) Color change of Probe **1** nanofiber membrane in the presence (left) and absence (right) of H_2_S vapor [29]. (**d**) Confocal fluorescence imaging of H_2_S in rice roots exposed to drought stress and the rice roots were exposed to drought stress for 0–7 h, then treated with the probe **3** (5 μM) for 1 h. Incubated under drought stress for 0 h (a1–a3), 1 h (b1–b3), 3 h (c1–c3), 5 h (d1–d3), 7 h (e1–e3), and 7 h + NEM (f1–f3) and then treated with the BSZ-H2S probe (5 μM) for 60 min. λ_em_ = 650–730 nm, and λ_ex_ = 633 nm. Scale bars = 100 μm [31]. (**e**) Probe paper used for detection of H_2_S produced by croaker stored at −20 °C (left) and 25 °C for 2 days (right), and photographs taken either directly after detection (left) or under UV light (λ_ex_ = 365 nm) (right) [36]. (**f**) Colorimetric and fluorescent photographs of probe **14** test paper under natural light and 365 nm UV irradiation with different concentrations of H_2_S [37].

**Figure 2:**
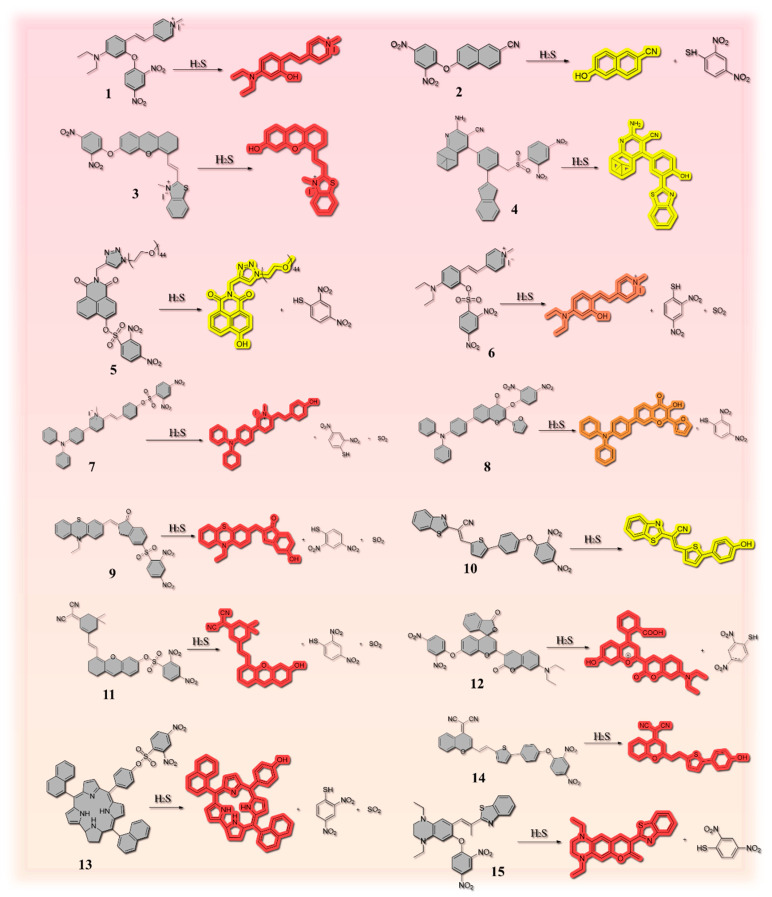
Various hydrogen sulfide fluorescent probes based on the thiolysis reaction of 2, 4-dinitrophenyl (DNP). The numbers on the diagram are used in place of the name of the compound.

DNP can trap H_2_S, so the reaction of this group with H_2_S can be well applied in the design of fluorescent probes. Under the nucleophilic effect of H_2_S, the DNP ether is thiolated by the H_2_S-based group, and the sulfation process is fully triggered, which is accompanied by a change in fluorescence intensity, and thus the fluorescent probe can respond (Figure 1a). At present, DNP-based fluorescent probes have been widely used for detecting H_2_S in food. However, different fluorophores and backbones can give different properties to the probes. The fluorescent probe **7** designed by Xie et al. (Figure 2) has triphenylamine and 2,4-dinitrobenzenesulfonyl groups incorporated into the styrylpyridinium scaffold, and the pyridinium salt in the scaffold can increase the solubility of the probe and its emission wavelength, which makes the probe mitochondrially targeted. In addition, the probe has a color change that appears in the “naked eye” state (Figure 1b), revealing the possibility of visualizing H_2_S content in food samples [27]. Probe **1** utilizes the excellent properties of high stability and large Stokes shift of 4-diethylaminosalicylaldehyde [28], so it was chosen as the fluorophore of probe **1**. It is worth mentioning that Zhong et al. also detected H_2_S in the gas phase and prepared nanofiber membranes doped with probe **1** by electrostatic spinning and exposed them to saturated H_2_S vapor. The results were obtained under daylight, as shown in Figure 1c, indicating that the fiber membrane was able to detect H_2_S in the gas phase, and the high porosity and large specific surface area of the membrane composed of electrostatically spun filaments could improve the sensitivity of the probe to some extent [29]. However, it remains to be seen whether this structured fiber membrane can be used for trace analysis in food. Surprisingly, test strips impregnated with probe **12** could be used for visual determination of H_2_S gas due to food spoilage [30]. Thus, this may support the design and establishment of a food spoilage assessment system in the future.

Compared with other H_2_S-specific fluorescent probes, probe **2** has a simple structure, is easy to synthesize, and achieves the detection of H_2_S in wine using a spiked recovery assay with recoveries of 90–103%. It has great potential for application in wine quality control and freshness assessment [31]. The structure may hold some promise for future rapid synthesis of probes for use in the detection field, but its application is not yet broad enough and there is still room for development. Compared to probe **2**, probe **3** uses benzothiazole to provide fluorescent signals. Meanwhile, this probe has been used for in situ imaging of rice in addition to real-time imaging of cells and zebrafish. A positive correlation between drought stress treatment and H_2_S levels was observed. The fluorescence intensity of the red channel increased with increasing drought stress treatment time (Figure 1d) [32]. However, probe **4** utilizes the nopinone-based cyanopyridine amine structure with stable optical properties [33], but adds the same benzothiazole moiety as probe **3** in the neighboring position of the hydroxyl group, which results in the ESIPT phenomenon, and the fluorescence ability can be greatly enhanced. In addition, probe **4** can be used for detecting H_2_S in a variety of food samples [34]. It is expected to provide some ideas for food quality analysis.

Probe **6** was constructed using N,N-diethyl-4-vinylaniline with a 1-methylpyridinium iodide moiety to form the SPy-OH fluorophore, and it was constructed to form a donor-π-acceptor (D-π-A) structure by combining N,N-diethyl-4-vinylaniline with a 1-methylpyridinium iodide moiety, which was chosen as the mitochondrial targeting moiety [38,39]. Also, in this case, the reaction of the DNS ester group was initiated by HS- via a nucleophilic reaction, releasing the Sy-OH fluorophore. Surprisingly, the probe was able to respond to H_2_S at an ultrafast rate of 4 s, which is a great advantage in detecting H_2_S content in food samples to infer the freshness of such foods [4]. Probe **14**, which has a similar response mechanism to probe **6**, releases a red fluorophore upon complete cleavage of its dinitrophenyl group moiety, resulting in fluorescence enhancement. Water quality can be detected and visualized in food samples (Figure 1f) [37].

Similar to probe **6**, probe **15** was constructed using a combination of 2-benzothiazole acetonitrile and 7-hydroxytetrahydroquinoxaline-6-carboxaldehyde in the D-π-A system, which was designed to extend the emission wavelength of the probe well [40]. DNP triggers the fluorescence burst of the probe via a photo-induced electron transfer (d-PET) process excited by the electron donor. Upon reaction with H_2_S, the hydrogen sulfide induces CeO bond cleavage of the probe. Subsequently, the resulting phenol salts attack the a,b-unsaturated nitrile moiety by intramolecular cyclization, resulting in a fluorophore. Since the fluorophore is highly conjugated, it restricts the rotation of the C=C double bond, causing the fluorescent probe to produce intense red fluorescence upon reaction with H_2_S [41]. A tandem reaction (thiolation-cyclization) has been proposed in the design, whereby more diverse NIR fluorescent probes can be designed by substituting the protecting group [42]. This point can give ideas to many researchers. A two-step tandem reaction initiated by HS- was used to construct probe **15**.

In addition, probe **8** is based on a natural flavonol and has been used to detect H_2_S in environmental water samples, beer, wine, milk, acid sprouts, and eggs [43]. However, probe **10** is based on (E)-2-(benzo[d]thiazol-2-yl)-3-(5-(4-hydroxyphenyl)thiophen-2-yl)acrylonitrile as the fluorophore, and due to the bursting effect of the DNP fragment on the fluorescence, probe **10** hardly fluoresces. However, in the presence of H_2_S, the recognition group is dissociated and the fluorophore (BAOH) is released, allowing the probe to respond and fluoresce yellow [44].

In general, researchers select stronger electron-absorbing groups for probe design. As an analog of DNP, the introduction of DNBS tends to enable the visualization of H_2_S under lower background signal conditions. Its strong electron-withdrawing ability accelerates the H_2_S-mediated thiolation reaction, further improving the response time. Therefore, in addition to DNP, DBNS is often used as a recognition group for H_2_S. Xu et al. selected phenothiazine derivatives as the fluorophore portion of probe **9** to make the probe with low cytotoxicity, easy modification, and good stability for more efficient, sensitive, and safe detection of H_2_S in food samples [45]. Xiao et al. designed fluorescent probe **5** by combining naphthalimide fluorophore; it was selected as the fluorophore of the probe due to the high chemical stability it imparts to the probe, its easy modification, and, most importantly, its low cost. The probe also achieved “naked eye” observation of the reaction state in real food samples (Figure 1e) [36], which will be useful for the future mass production of the probe and its application in practice.

The design concept of Probe **11** is somewhat distinct from that of other probes. The 2,4-dinitrobenzenesulfonate (DNBS) group serves two distinct roles in the probe: it acts as the corresponding site and the electron acceptor. The fluorescence is extinguished by the photoelectron transfer effect (PET). The probe is essentially non-fluorescent due to the the PET effect, which is eliminated with the addition of H_2_S as a result of the reaction between the sulfonate bond and H_2_S. Once more, red fluorescence was observed as a result of the restoration of the intramolecular charge transfer effect (ICT) [46]. Probe **13** is a novel corrole-based fluorescent probe that exhibits sensitivity and can be employed to detect water quality. When the 2,4-dinitrobenzenesulfonate ester bond of probe 13 reacted with H_2_S and was cleaved, the foregoing PET effect was terminated, resulting in a bright red fluorescence signal [47].

Both DNP and DNBS are commonly utilized H_2_S recognition molecules for fluorescent probes. As illustrated in Table 1, most probes with recognition molecules DNP and DBNS exhibit a rapid response capability, enabling them to respond swiftly. Given that food is susceptible to environmental influences during storage, an inappropriate storage environment can result in accelerated spoilage. This characteristic of the probe, coupled with its rapid response time, enables the detection of H_2_S in food, thus determining its freshness. This type of fluorescent probe can, therefore, play a pivotal role in the assessment of food preservation.

**Table 1:** The main information of the probes detecting H_2_S in food samples.

Number	Response Time	LOD	Stokes Shift	Fluorescent Chromophore	H_2_S Reporter	Application	Ref.
1	/	10.5 µM	111 nm	4-diethylaminosalicylaldehyde	2,4-dinitrophenyl (DNP)	Red wine and beer	[29]
2	30 min	76 nM	/	6-hydroxy-2-naphthonitrile	2,4-dinitrophenyl (DNP)	Red wine and beer	[31]
3	/	104 nM	/	benzothiazole	2,4-dinitrofluorobenzene	Rice and lake water	[32]
4	/	79 nM	/	nopinone	2,4- dinitrobenzenesulfonyl ester group	River water, lake water, stream water, red wine, beer, hen egg, duck egg, quail egg, and pigeon egg	[34]
5	2 min	9.95 nM	/	naphthalimide	dinitrobenzenesulfonyl	Fish	[36]
6	3 min	41.95 nM	111 nm	combining N, N-diethyl-4- vinylaniline with a 1-methylpyridinium iodide moiety	2,4- dinitrobenzenesulfonyl group	Beer	[4]
7	3 min	41.9 nM	/	styrylpyridinium scaffold	2,4-dinitrobenzenesulfonyl chloride	Chicken, pork, beef	[27]
8	3 min	96 nM	210 nm	natural product flavonol	2,4-dinitrophenyl (DNP)	Red wine, beer, eggs, milk, and sour bamboo shoots	[44]
9	Within 90 s	0.14 μM	220 nm	phenothiazine derivative	2,4-dinitrobenzene sulfonyl chloride	Pork, chicken, beef and fish	[36]
10	Within 90 s	76 nM	145 nm	benzothiazole derivative	2,4-dinitrophenyl (DNP)	Pork, chicken and shrimp	[45]
11	60 s	1.27 μM	190 nm	(E)-2-(3-(2-(6-hydroxy-2,3-dihydro-1H-xanthen-4-yl)vinyl)-5,5-dimethylcyclohex-2-en-1-ylidene)malononitrile	2,4-dinitrobenzenesulfonyl ester group	Chicken, eggs, and fish	[47]
12	/	35.70 nM	/	functional coumarin-benzopyrylium platform (FC-OH)	2,4-dinitrophenyl moiety	Pork, chicken and shrimp	[30]
13	10 s	61 nM	/	5,15-bis(naphthyl)3-10-(4-hydroxylphenyl) corrole (NPC–OH)	2,4-dinitrobenzenesulfonyl group (DNBS)	Chicken, beef, pork, cracked egg and fish	[35]
14	Within 30 s	58 nM	175 nm	dicyanomethylene-4H-pyran (DCM)	2,4-dinitrophenyl group	Shrimp, pork, and chicken	[37]
15	/	38.30 nM	126 nm	1,4-diethylpiperazine-modified iminocoumarin-benzothiazole	2,4-dinitrophenyl group	River water and red wine	[44]
16	6 min	0.44 μM	220 nm	β-diketone boron difluoride complex	C=C bonds	Red wine	[48]
17	50 s	19.43 nM	/	α-Pinenecombined with imidazole ring	C=C bonds	Pork, fish, and shrimp	[10]
18	/	0.22 µM	/	benzo-hemicyanine	C=C bonds	Egg, raw meat and fish	[49]
19	/	0.98 μM	/	coumarin dye	C=C bonds	Pork, chicken sample, and garlic sample	[50]
20	30 s	99.68 nM	/	benzothiazole	C=C bonds	Beer	[51]
21	/	0.37 µM	/	combined the naphthalimide with a morpholine moiety	7-nitro-1,2,3-benzoxadiazole (NBD) amines	Beer	[52]
22	/	18 nM	/	coumarin	thenoic acid	Red wine	[53]
23	/	54 nM	/	/	quinolinium-phenol vinylic conjugate	Eggs and pork	[54]
24	/	80 nM	205 nm	coumarin2212dicyanoisophorone conjugate	7-nitro-1,2,3-benzoxadiazole (NBD)	Pork, shrimp, and eggs	[55]
25	Within 5 s	87.5 nM	147 nm	/	the alkenyl group	Beef, shrimp	[56]
26	/	0.10 mM (S/N = 3)	/	naphthofluorescein	thiophenecarboxylic ester	Red wine	[57]
27	Within 10 s	4.3 nM	/	6,8-dichloro-7-hydroxy-9,9-dimethylacridin-2(9H)-one	oxygen-nitrile bond	Crucian chicken, shrimp, pork and egg	[58]
28	/	54 nM	/	merocyanine	2-thiophenecarbonyl group	Beef, pork, and chicken	[59]
29	Within 8 min	34 nM	/	cyanine derivative	phenyl chlorothionocarbonate	Pork and shrimp	[60]
30	Within 3 min	0.144 µM	/	derivative of Indocyanine green (ICG)	Cu^2+^	Red wine, beer, meat, milk, and sweet potato	[61]
31	10 min	56 nM		pyren-1-amine	azido group	Red wine	[62]
32	/	8.12 µM	/	/	Two double bonds between Calix[4]arene and methylpyridinium iodide fragments	Beef and apricot seeds	[63]

### 2.2. Based on Double Bond Addition

**Scheme 2:**
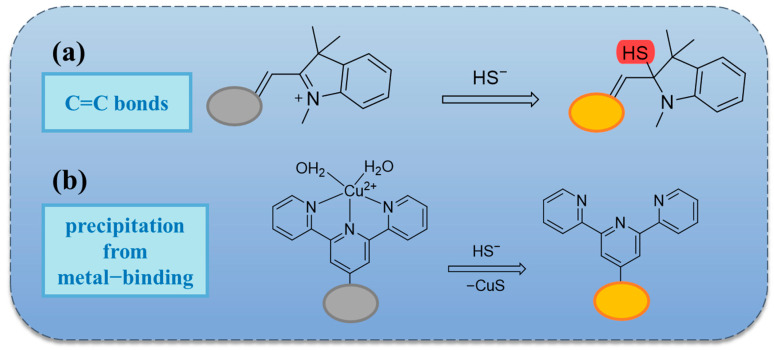
Approximate response mechanism of (**a**) double bond addition and (**b**) precipitation from mental binding [64].

**Figure 3:**
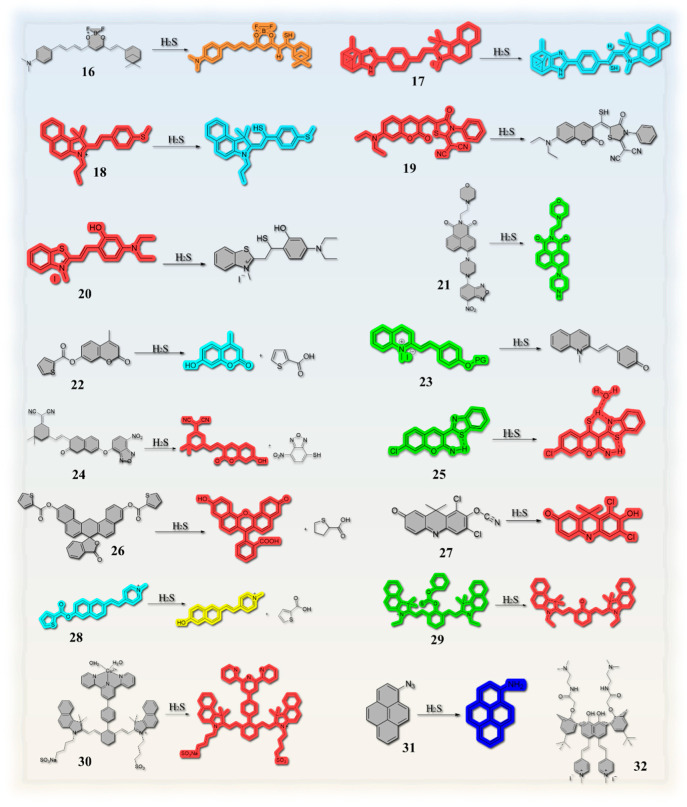
Various fluorescent probes for the detection of hydrogen sulfide in food samples. The numbers on the diagram are used in place of the name of the compound.

**Figure 4:**
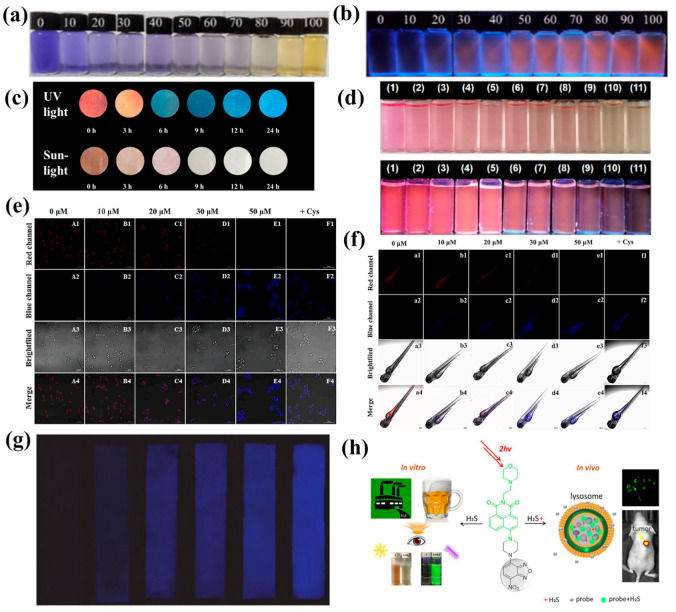
Photographs of Probe **16** solutions with 0–100 μM H_2_S added under (**a**) daylight and (**b**) 365 nm UV light [48]. (**c**) Changes over time (0–24 h) in a 0.1 M H_2_S solution when a filter strip loaded with probe **17** is exposed to UV light (top) and sunlight (bottom) [10]. (**d**) Colorimetric and fluorescent colorimetric recognition of H_2_S (0 µM, 12 µM, 24 µM, 36 µM, 48 µM, 60 µM, 72 µM, 84 µM, 96 µM, 108 µM, and 120 µM, respectively) in Tris-HCl buffer at pH = 7.4 by probe **19** (10 µM) [50]. (**e**) RAW264.7 cells were incubated with (A1–E4) TIBI (15 µM, 0.5 h); (B1–B4) H_2_S (10 µM, 20 min); (C1–C4) H_2_S (20 µM, 20 min); (D1–D4) H_2_S (30 µM, 20 min); (E1–E4) H_2_S (50 µM, 20 min); (F1–F4) Cys (0.1 mM, 6 h) and TIBI (20 µM, 0.5 h). λ_ex_ = 405 nm, λ_em_ = 500–550 nm (blue channel), λ_ex_ = 600 nm, λ_em_ = 650–750 nm (red channel), scale bar = 50 µm [10]. (**f**) Confocal fluorescent photographs of zebrafish were incubated with (a1–a4) TIBI (15 µM, 0.5 h); (b1–b4) H_2_S (10 µM, 5 min); (c1–c4) H_2_S (20 µM, 5 min); (d1–d4) H_2_S (30 µM, 5 min); (e1–e4) H_2_S (50 µM, 5 min); (f1–f4) TIBI (20 µM, 0.5 h) and Cys (0.1 mM, 2 h). λ_ex_ = 405 nm, λ_em_ = 500–550 nm (blue channel), λ_ex_ = 600 nm, λ_em_ = 650–750 nm (red channel), scale bar = 200 µM [10]. (**g**) Change in fluorescence of test paper to detect H_2_S in red wine samples with increasing H_2_S concentration from left to right. A handheld UV lamp was used to excite the paper at 365 nm [62]. (**h**) Response mechanisms and applications of probe **21** [52].

The addition of H_2_S to a C=C double bond results in a Michael addition reaction, which effectively disrupts the original compound’s conjugated structure (Scheme 2a). This process ultimately leads to the emission of fluorescence. With this strategy, Shen et al. synthesized probe **16** (Figure 3). The probe changed color from blue to yellow when observed with the naked eye (Figure 4a), and a change in fluorescence from no fluorescence to orange fluorescence was observed under a fluorescence microscope (Figure 4b). The probe exhibits excellent photostability, maintaining a stable fluorescence intensity for at least 48 h [49]. In contrast, probe **17** designed by Xu et al. (Figure 3) employs a nucleophilic addition of H_2_S in the olefinic portion, which results in the disruption of π-conjugation and the blocking of intramolecular charge transfer (ICT). The switching transition of the ICT process gives rise to a change in fluorescence. The probe exhibits a color change from reddish brown to white when observed visually, and under fluorescence microscopy, the color shifts gradually from orange-red to blue (Figure 4c). Furthermore, the probe demonstrated excellent optical response characteristics in the detection of trace H_2_S in living cells (Figure 4e) and in zebrafish (Figure 4f) [10]. It is noteworthy that probe **18** designed by Magesh et al. in 2023 (Figure 3) is capable of simultaneous detection of CN- and HS- due to the electron-deficient nature of benzo-hemicyanine and the presence of electron-rich sulfur atoms in 4-(methylthio)benzaldehyde. This facilitates a donor between the polar C=C double bonds, thereby generating donor-π-acceptor interactions. CN- and HS- are capable of undergoing selective nucleophilic addition reactions at their respective reaction sites, which results in alterations in fluorescence [50]. The aforementioned fluorescent probes are suitable for use in the detection of food samples.

In contrast to other fluorescent probes that have been synthesized based on the C=C double bond mechanism, probe **19** exhibits an “ON–OFF” fluorescence response. The ICT effect is disrupted by the Michael addition of H_2_S to the C=C double bond, which results in the loss of fluorescence [51]. The mechanism of probe **20** is essentially analogous to that of probe **19**, with the fluorescence response also occurring in an “ON–OFF” manner. The probe is employed to analyze water and beer samples [62].

### 2.3. Thiolation Reactions Based on 7-Nitrobenzo-2-oxa-1,3-diazole (NBD)

**Scheme 3:**
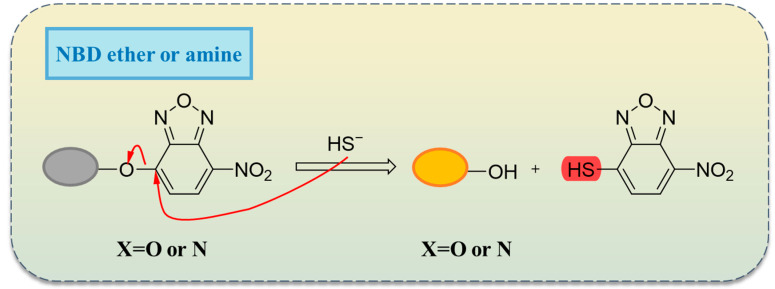
Approximate response mechanism of NBD ether or amine [46].

The thiolysis of NBD ethers or NBD amines is triggered by H_2_S via nucleophilic aromatic substitutions, resulting in the release of fluorophores (Scheme 3). The fluorescent probe **21** (Figure 3), designed by Luo et al., exhibits a double PET effect. When the morpholine moiety acts as an electron donor, the electrons on it are able to be transferred to the naphthylimide core, thereby triggering the α-PET effect. In this process, the electrons flow from the electron-rich donor (morpholine) to the electron-deficient acceptor (naphthylimide). Concurrently, this electron flow indirectly generates further interactions between the naphthimide core and another electron-deficient fragment (NBD), thereby enhancing the overall photoinduced electron transfer phenomenon. It is noteworthy that, in addition to this, the d-PET effect coexists in the system. The combination of these two dual effects allows probe **21** to produce minimal autofluorescence under acidic conditions, thereby facilitating optimal optical imaging. Furthermore, the probe exhibits a visible color change from orange to light yellow and a shift in fluorescence emission from no fluorescence to green fluorescence. The probe is capable of detecting changes in the endogenous and lysosomal levels of H_2_S in tumor cells in living systems. Moreover, the probe was successfully employed for the detection of H_2_S in environmental wastewater and food samples, including beer (Figure 4h). However, probe **24** (Figure 3) exhibits a slightly different mechanism than probe **21**. Additionally, probe **24** is an open fluorescent probe. Probe **21** employs the coumarin 2212-dicyanoisophorone conjugate as a fluorophore, with NBD serving as the specific recognition site for H_2_S. When the fluorophore is attached to NBD through ether bonding, the electron-donating ability of the hydroxyl group is constrained, thereby inhibiting the ICT effect and resulting in a rapid increase in fluorescence. Upon adding H_2_S, the NBD undergoes partial cleavage, releasing the fluorophore. Consequently, the probe’s fluorescence was restored following the completion of the reaction [55].

### 2.4. Reactions Based on Azide Groups

**Scheme 4:**
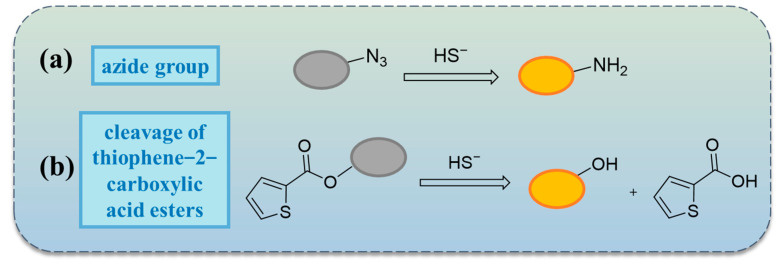
(**a**) Approximate response mechanism of azide group. (**b**) Approximate response mechanism of cleavage of thiophene-2-carboxylic acid esters [35].

The azide group has been demonstrated to possess excellent reducing capabilities, which can effectively convert the electron-withdrawing azide group into an electron-donating amino group (Scheme 4a). This process serves to restore the fluorescence that was previously extinguished by the azide group. Based on this, probe **31** (Figure 3) was designed and applied to the detection of H_2_S in red wine samples. As illustrated in Figure 4g, the test strips display enhanced fluorescence intensity (dark blue) in response to elevated H_2_S concentrations within the fluorescence field. This suggests that the probe exhibits excellent capability for detecting authentic food samples, with a detection limit as low as 1 μM for red wine samples [62].

### 2.5. Others

**Figure 5:**
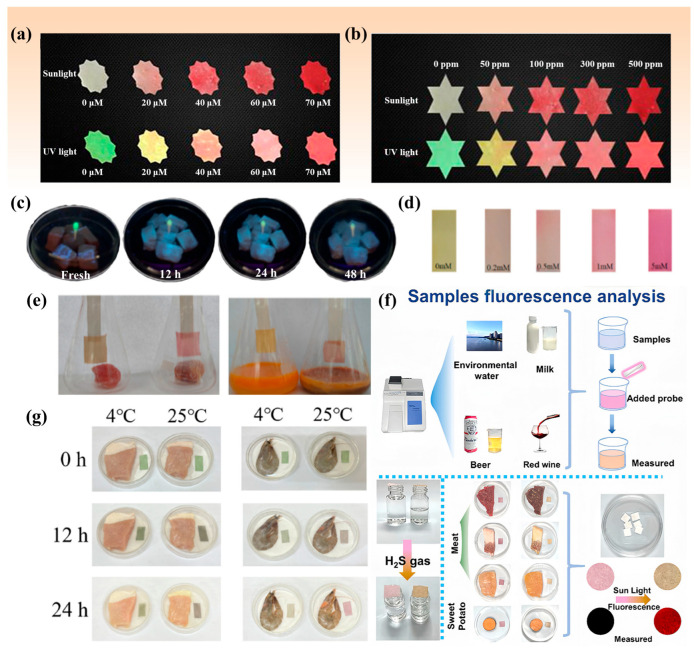
(**a**) Images of probe **25**-based test paper for NaHS detection in sunlight (up) and under 365 nm UV light (down) [56]. (**b**) Image of probe **25**-based test paper for the detection of H_2_S gas under 365 nm light [56]. (**c**) The color of beef test paper changes over time in long-wave light [63]. (**d**) Color change of the detection strip loaded with probe **23** for different concentrations of H_2_S in ambient light [54]. (**e**) Detection strips of probe **23** for the detection of H_2_S produced during the processing of pork (left) and eggs (right). Each group was stored at −4 °C (left) and 25 °C (right), respectively [54]. (**f**) Probe **30** is based on the copper ion burst and substitution mechanism and its near-infrared fluorescence detection of H_2_S [61]. (**g**) The test paper of probe **29** monitors the phenomenon of H_2_S production and its color change during spoilage of pork (left) and shrimp (right) [60].

It has been demonstrated that molecules exhibiting AIE properties are capable of maintaining high light stability and robust fluorescence emission in the polymerized state [65]. Probe **25** designed by Wang et al. (Figure 3) is an AIE ratiometric probe, which is capable of accurate analysis in food safety testing based on the acquisition of the ratio of the intensities of the two emission peaks of a fluorescent probe as well as its unique response products. In a 70% ethanol aqueous solution, the addition of hydrogen sulfide (H_2_S) results in the formation of new hydrogen bonds between the N and H atoms of the N-H-S moiety. The formation of new hydrogen bonds can further enhance the rigidity of the probe **25**. Furthermore, the formation of hydrogen bonds requires the participation of water molecules. The formation of hydrogen bonds facilitates the transition of probe AIE from a green to a red aggregated state. In essence, the addition of H_2_S gas induces a transformation from the aggregated green state of rod AIE to a globular red state (Figure 5a,b). This probe is also suitable for trace analysis [56].

In contrast to probe **25**, probe **26** (Figure 3) employed a thiophenecarboxylic ester as the response unit for H_2_S, while naphthofluorescein was utilized as the fluorescent signaling group. The low fluorescence intensity of probe **26** was due to the protection of the probe’s hydroxyl group by the thiophenecarboxylic ester. The release of the phenolic hydroxyl group upon treatment with an excess of H_2_S restores the structure of the fluorescent signaling moiety and enhances the fluorescence intensity [57]. Although it is capable of detecting the concentration of H_2_S in red wine samples, the fluorescence intensity can only be significantly enhanced after treatment with excess H_2_S. As a result, it is challenging to achieve rapid detection in practical applications, and its range of applications is limited. Probe **22** (Figure 3), which employs a similar mechanism to probe **26** (Scheme 4b), is also based on the formation of 7-hydroxy-4-methylcoumarin through the disruption of the thiophenate ether group in the probe, which is induced by H_2_S. This process results in an enhanced fluorescence intensity, as illustrated in Figure 3. This method is suitable for the determination of H_2_S levels in not only red wine but also beer [53].

Furthermore, based on the principle that H_2_S can trigger the release of protective groups (PG), Hu et al. introduced a quinoline–phenol vinyl conjugate to design a fluorescent probe. The release of PG by H_2_S results in the formation of unstable amphiphiles (QL-Oi), which subsequently undergo self-incineration and ultimately give rise to a non-fluorescent neutral quinoline–phenol vinyl conjugate. The two daylight color changes with high contrast (Figure 5d) facilitate a clear determination of food freshness and offer new possibilities for solid-state paper test experiments with H_2_S in real food samples (Figure 5e) and food freshness assessment [54].

In the presence of H_2_S, the oxygen–carbonitrile bond in DDAO-CN is rapidly hydrolyzed, releasing the fluorophore 1,3-dichloro-7-hydroxy-9,9-dimethylacridin-2(9H)-one (DDAO), which results in a change of the solution from yellow to blue. This resulted in an on-type fluorescent response, exhibiting intense red fluorescence under UV light irradiation [58]. However, probe **29** (Figure 3) is composed of a novel electron-available phenyl thiochlorocarbonate, which inhibits fluorescence by preventing charge transfer within the molecule. In the presence of H_2_S, the electron donor is removed, thereby restoring the intermolecular charge transfer (ICT) effect and resulting in fluorescence. Meanwhile, during the detection of the food sample, the color of the test strip undergoes a gradual transition from green to red (Figure 5g) [60]. The introduction of H_2_S to probe solutions 27 and 29 produces a gradual alteration in solution color or test strip color, respectively, resulting in a markedly contrasting and readily discernible hue that differs significantly from the original. This enables the tracing of H_2_S produced during the deterioration of foodstuffs.

In particular, probe **28** (Figure 3) is a dual-channel fluorescent probe that exhibits selective recognition of H_2_S. The fluorophore of the mercapto compound and the reactive site of 2-thiophene carbonyl enable the specific recognition of H_2_S. The removal of thiophenecarboxylic acid by the addition of H_2_S results in the release of the yellow dye T-B and enhances the ICT effect [51], which is responsible for the observed fluorescence changes. It is noteworthy that the reaction must be conducted in an aqueous solution both before and after, which has a deleterious impact on the expeditious detection of H_2_S gas produced during the spoilage of food samples.

In addition to the aforementioned design concepts, probes may also be constructed through the principles of copper ion bursting and substitution (Scheme 2b). For example, probe **30** (Figure 3) is based on this principle. The fluorescent molecule IR820, a derivative of indocyanine green (ICG), is employed as the fluorophore, and the probe’s fluorescence is triggered following the chelation with copper ions. Following the reaction with H_2_S, the fluorescence intensity is restored. The probe demonstrated an effective and discernible fluorescence response in detecting environmental water and food samples (Figure 5f). Probe **32** (Figure 3) is more complex in structure but has the capacity for CN^-^ and HS^-^ detection, is versatile, and the detection in fresh beef samples may be visualized under fluorescent conditions (Figure 5c). This could prove a valuable tool in detecting food safety issues [63].

## 3. Methods for H_2_S Detection in Foods

At a glance, there are three principal methods for the detection of H_2_S in real food samples using fluorescent probes. At present, the primary objective is the utilization of test strips for the visualization of H_2_S content through coloration, a technique that is predominantly employed for the detection of foodstuffs such as beef, chicken, pork, eggs, and shrimp. The second method is the spiked recovery test, which is typically employed for the detection of red wine, beer, and other similar beverages.

### 3.1. Colorimetric Method

**Figure 6:**
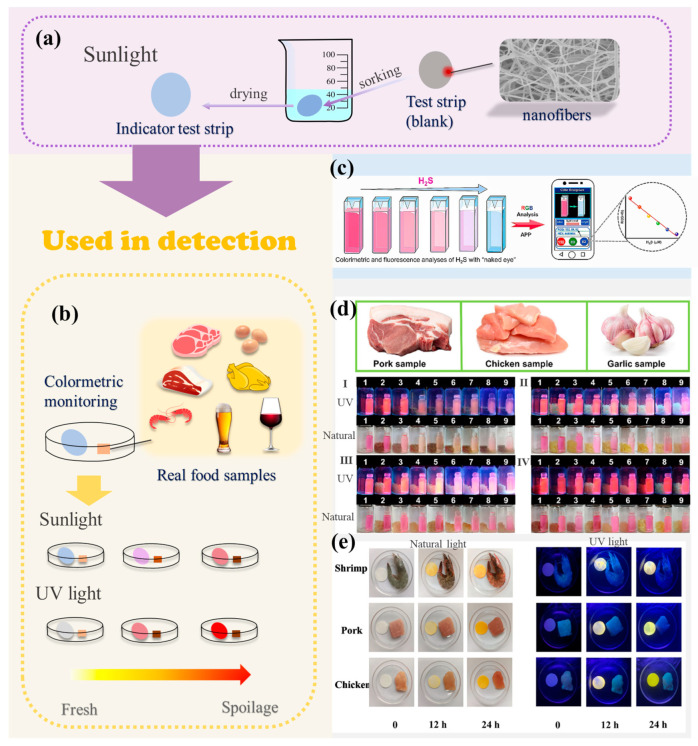
(**a**) Preparation of test strips loaded with H_2_S fluorescent probes. (**b**) Colorimetric detection of H_2_S fluorescent probes. (**c**) H_2_S in actual samples was quantitatively detected by smartphone [50]. (**d**) Colorimetric and fluorescence colorimetric response of the probe **19** to H_2_S produced during the spoilage of three food products. (I) Pork sample. (II) Garlic sample. (III) Chicken sample. (1) XDS (10 μM) solution was preserved in the dark at room temperature for 7 days. (2–9) Colorimetric and fluorescent colorimetric images of XDS (10 μM) solutions when foodstuff samples were preserved in the dark at room temperature for 0 day, 1 day, 2 days, 3 days, 4 days, 5 days, 6 days and 7 days, respectively, under natural light and UV light (365 nm). (IV)(1–3) XDS (10 μM) solution and pork sample were coexisted in refrigerator at 5 °C for 1 day, 3 days and 7 days, respectively; (4–6) XDS (10 μM) solution and chicken sample were coexisted in refrigerator at 5 °C for 1 day, 3 days and 7 days, respectively; (7–9) XDS (10 μM) solution and garlic sample were coexisted in refrigerator at 5 °C for 1 day, 3 days and 7 days, respectively [50]. (**e**) Colorimetric and fluorescence photographs of test paper and food samples from Probe **10** under natural light and UV (365 nm) illumination [44].

The most prevalent colorimetric method employed in the present study is the test strip method. The rationale behind the design of the test strip is to employ a straightforward physical deposition technique to load a fluorescent probe onto a test strip [66], thereby enabling the strip to respond to the H_2_S [67] that is generated during the deterioration of the foodstuff (Figure 6a,b). The method primarily employs the use of filter paper sheets, which are soaked in the probe solution and subsequently air-dried to create filter paper sheets that are loaded with the probe, thereby producing test strips. The freshness of the food can be determined by certain color changes [61]. Cai et al., in 2024, designed a turn-on fluorescent probe **10** with a large Stokes shift, a short response time, and a low detection limit. The probe was tested on real food samples (shrimp, pork, and chicken) and showed a gradual deepening of the yellow color over time (0 h, 12 h, and 24 h) (Figure 6e).

Furthermore, as illustrated in Figure 6c, they devised a smartphone-based analytical approach. As the concentration of H_2_S increased, the intensity of the yellow fluorescence also increased. The H_2_S content of the product was calculated from a linear relationship graph after the G/B value was determined by a color recognition program on the smartphone under UV light conditions. This value was then converted to a specific RGB value [44]. This application provides a portable and prospective strategy with significant potential for quantitative analysis of H_2_S in food preservation. Furthermore, food testing applications employ the probe **19** solutions to specifically respond to H_2_S. This includes the “ON–OFF” type fluorescent probe designed by Shang et al. As the time is prolonged, the more severe the food spoilage, the lighter the color of the probe solution in the vial. Fluorescence intensity is observed to be weaker when examined under fluorescent conditions (Figure 6d) [50]. Conversely, the test strip method may offer greater convenience in terms of detection, and the test strips are more portable. Furthermore, it is capable of visualizing the freshness of food samples and assisting in evaluating the efficacy of preservation methods.

Nevertheless, current research has yet to identify test strips that can achieve a “naked eye” response in a short period and be applied on a large scale to detect H_2_S in real samples in daily life. It is necessary to conduct further research to determine whether the color recognition program on a smartphone can be detached from the fluorescent field and accurately recognized in daylight. It is also important to note that the test strips or probe solutions must be placed near the food product to obtain an accurate response. Additionally, further investigation is required to ascertain whether this procedure affects the safety of the food product. Therefore, further experimental verification is required.

### 3.2. Spiked Recovery Test

The spiked recovery test is typically employed to identify the presence of red wine, beer, and water quality samples. These samples possess distinctive colors that can intensify the background hue of the test paper, potentially obscuring the results. Accordingly, the spiked recovery test is typically employed to detect the presence of hydrogen sulfide. Following the measurement of the hydrogen sulfide content, a comparison is made between the measured value and the standard to ascertain whether the concentration of hydrogen sulfide in the food sample exceeds the specified limit. If the standard limit is exceeded, the food in question is rendered unfit for human consumption or commercial use. In 2017, Wang et al. designed probe **22** for the detection of hydrogen sulfide in red wine and beer. The results demonstrated that the recoveries for the detection of H_2_S in wine ranged from 91.2 to 110.0%, and were validated by methylene blue spectrophotometry, effectively illustrating the feasibility of the method [53]. In 2018, Probe **2** was also designed to detect hydrogen sulfide in the same sample. The recovery for red wine was superior to that observed in 2017, with values ranging from 90.0% to 103.0% [31]. Given the extensive existing data on the efficacy of fluorescent probes for the precise detection of hydrogen sulfide in authentic food samples (including water, red wine, and beer), no validation experiments for the methylene blue spectrophotometric method have been identified as having been conducted in recent years. In 2023, Xie et al. designed probe **20** to detect hydrogen sulfide in beer, with recoveries ranging from 95.6% to 103.7%. It was compatible with the determination of the H_2_S recovery concentrations, which were matched [51]. This suggests that the technology of fluorescent probes is becoming increasingly sophisticated, with recoveries approaching the established standard values. Xu et al. employed three water samples (tap water, Black Lake Spring, and Daming Lake) to detect hydrogen sulfide, yielding detection recoveries that ranged from 91 to 112%. Notwithstanding slight discrepancies in the recoveries. However, they made a noteworthy observation in the paper regarding the application of probe detection. According to the GB11607-1989 [68], the concentration of H_2_S in water should be less than 0.2 mg/L. The detection limit of this probe in water quality is 5.73 μM, which demonstrates that the probe NBT can detect the hydrogen sulfide content in water quality [69].

## 4. Conclusions and Outlook

In recent years, an increasing number of studies have revealed that H_2_S is not only a key gaseous signaling molecule in living organisms but also a key molecule that is indispensable in the process of maintaining redox homeostasis. The intricate functions and significance of this gas have prompted a reexamination of its role in biological processes, offering novel insights into its involvement in physiological regulation and pathological states. Furthermore, H_2_S is inextricably linked to most high-protein foods. In the contemporary era, the gradual development of the Internet and food science and technology has led to a heightened focus on food safety and quality issues among the general public. Consumers have a heightened preference for food that is fresh. In order to maintain optimal freshness and maximize shelf life, effective measures to prevent food spoilage are essential and have become a significant concern in the food industry. In the contemporary food industry, the presence of H_2_S is inextricably linked to a diverse array of high-protein foods. This substance, renowned for its distinctive volatile properties, serves as a crucial indicator of the freshness of these foods. Fluorescent sensors are proving an effective solution to this problem. The use of high-performance fluorescent sensors offers a rapid and low-biological-destructive method of monitoring, preventing, and reducing food spoilage caused by H_2_S. If the threshold is exceeded, prompt action can be taken to avert potential food safety concerns, thereby guaranteeing that consumers have access to safe and wholesome food.

**Figure 7:**
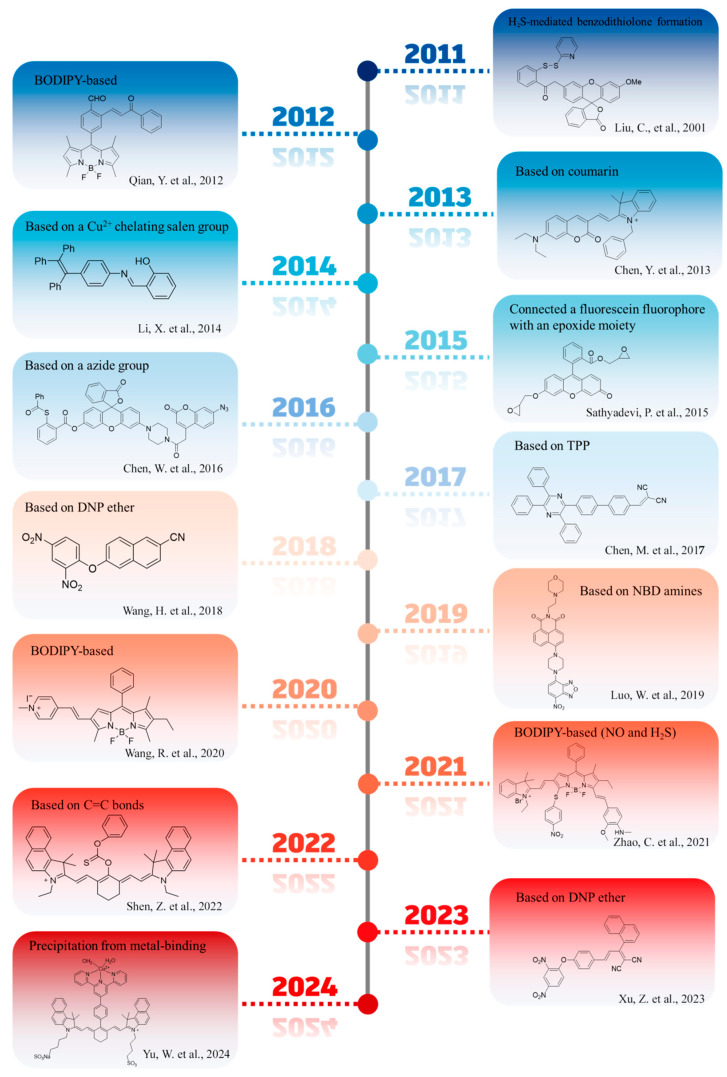
The development of the fluorescent probe of H_2_S [23,31,48,52,61,71,72,73,74,75,76,77,78,79].

**Figure 8:**
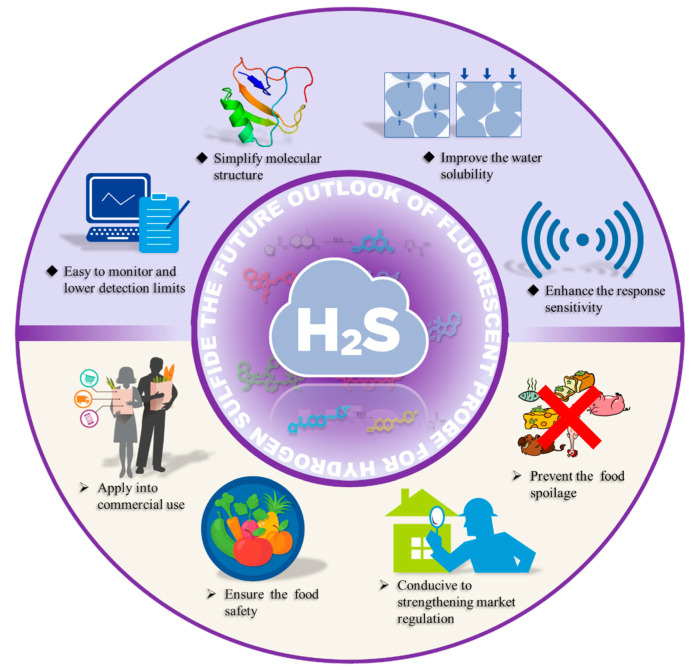
The future outlook of the fluorescent probe of H_2_S.

Currently, most fluorescent probes designed for use with real samples are oriented towards applications such as the thiolation of DNP, Michael’s addition of C=C double bonds, and thiolation of NBD, among others. The incorporation of a fluorophore renders the probes highly efficacious (Figure 7). Several high-performance probes with low detection limits and low response times have been developed, including probes **13**, **25**, and **27**, among others. Some probes are fully water-soluble, such as probe **32**, but their structures are complex. Simultaneously, probes such as probe **2**, which are simple and rapid to synthesize, are worthy of greater respect. However, they lack sufficient rapidity and versatility in their applications. In contrast, probe **4** has a broad range of applications and a multitude of variants. It can thus be concluded that, despite the design and synthesis of numerous fluorescent probes with excellent performance for detecting H_2_S, these probes still exhibit some limitations. In recent years, fluorescent probes have been developed that impede the ESIPT process by masking the phenolic hydroxyl groups with detection groups, thereby facilitating the detection of H_2_S [70]. This concept serves as a crucial reference point for the development of novel fluorescent probes. The advancement of novel fluorescent probes for the detection of H_2_S in foodstuffs must address the following criteria (Figure 8):

(1) It remains unclear whether it is possible to achieve complete water solubility without the involvement of organic reagents, which is still the case with only a few probes.
(2) The question thus arises as to whether it is possible to achieve complete water solubility and to detect gaseous H_2_S.
(3) The objective is to ascertain whether it is feasible to observe a change in color for trace amounts of H_2_S. This would entail enhancing the sensitivity and color change of the probes for small concentrations.
(4) The objective is to ascertain whether a color change can be observed for trace amounts of H_2_S. In other words, the sensitivity of the probe must be enhanced for small concentrations, and it must be determined whether the color change is affected by the concentration of H_2_S. Also, it is essential to pursue continuous improvements in the loading capacity of the probe on the test strip.

The extensive use of excellent NIR fluorophores such as BODIPY and coumarin, as well as signal molecules such as azide and 2,4-nitrophenyl ether, helps improve the water solubility and sensitivity of the probe. As fluorescent probes continue to be researched and improved, it is anticipated that they will not only remain at the scientific research level but also be utilized in real-life applications, playing an increasingly pivotal role in food safety supervision and food preservation assessment.

## Data Availability

No new data were created or analyzed in this study.
